# Corporate reconstructions of federal macroeconomic government institutions compared: USA then, Europe Now

**DOI:** 10.1057/s41295-021-00239-4

**Published:** 2021-03-24

**Authors:** Christakis Georgiou

**Affiliations:** Global Studies Institute, University of Geneva, Sciences II, Quai Ernest-Ansermet 30, CH - 1211 Genève 4, Switzerland

**Keywords:** Corporate reconstruction, Central banking, Fiscal federalism

## Abstract

This paper is a contribution to the comparative historical literature on the development of American and European federal macroeconomic government institutions, spawned by the 2010–12 Eurozone crisis. The literature has two major shortcomings, namely a lack of agreement on relevant periodization and a lack of causal explanations about the sources of the processes being compared. My claim is that the most relevant comparison is between the first half of the twentieth century for the USA and the period beginning in the 1980s for the European Union. The reason for this is the underlying and profound socio-economic process of the corporate reconstruction of American and European capitalisms, which I identify as the root cause of the development of federal macroeconomic government institutions in both polities.

## Introduction

Since 2010, the debate on fiscal federalism has taken centre stage in political economy debates about the EU. The Eurozone crisis highlighted the shortcomings of a monetary union lacking a lender of last resort, a substantial central budget with common debt and fiscal transfers from flourishing to struggling regions. As such, it acted as a “critical juncture”: European leaders had to decide in the midst of a frenetic crisis whether the Eurozone had to move backwards and disintegrate or rather take a leap forward and gradually graduate into a complete “fiscal union”.

In the event, the choice was made to take the leap. The initial steps in that direction were to allow the ECB to act as a lender of last resort for member states (De Grauwe 2013), thus ushering in “fiscal integration by default” (Schelkle 2013) through the fiscal mutualization operated via the central bank’s balance sheet. A European Stability Mechanism was also set up to lend to countries that had lost market access, but schemes for it to buy bonds on the markets never materialized, meaning that the ECB was left to do the heavy-lifting of underwriting member-state debts.^1^ By early 2021, the ECB held around 33% of outstanding Eurozone sovereign debt (Amundi 2021).

These initial steps were enough to stabilize sovereign bond markets, but since 2012 a debate has been ongoing about further steps, including a European investment budget and unemployment insurance scheme. The election of Emmanuel Macron to the French presidency in 2017 reignited the debate after he proposed a Eurozone budget in his Sorbonne speech (Chassany 2017), although the subsequent negotiations did not get very far.

Then came the huge economic crisis triggered by COVID-19 and the ball started rolling again. After months of negotiations, the European Council and Parliament agreed on a revised version of the Commission’s proposal to set up the Next Generation EU scheme, which together with the unemployment reinsurance facility SURE are expected to lead to 850 billion euros in bonds issued by the Commission and backed by the EU budget over the next few years, thus making the Commission one of the four biggest sovereign borrowers in Europe.

### The scholarly debate on the Eurozone’s evolution

The political science literature spawned by these developments is vast. Most of it builds on the economists’ analysis of the crisis as a balance of payments crisis and can be divided into two main strands, comparative and international political economy (good round-ups include, respectively, Nölke 2016 and Iversen et al. 2016; Frieden et al. 2017). CPE is mainly concerned with how the variety of national political economic institutions fuelled macroeconomic imbalances and prevented their swift resorption. The authors in this tradition generally find it difficult to conceive of the Eurozone in terms of political and institutional development as their approach tends to see national institutions as immutable, not as constantly evolving political-economic equilibria.

The IPE strand tends to think in terms of the international politics of balance of payments crises and macroeconomic adjustment. The natural reflex of IPE scholarship is thus to revert to intergovernmental bargaining power interpretations and to try to answer questions such as how were adjustment burdens distributed between and within states.

This is inadequate on two counts. First, it mischaracterizes the nature of the political process underway. As Frieden and Walter acknowledge, the Eurozone crisis was unique among balance of payments crises due to the institutional setting in which it took place. Crucially, unlike other such crises, it gave rise to the significant institutional innovations mentioned above that have an unmistakeable federation-building dimension about them. As a result, my claim is that the Eurozone needs to be understood through the lens of the political history of an evolving federal polity (“political development”), not that of International Relations.^2^

Second, IPE treatments fail to identify what I claim was the key conflict that shaped the course of events (Georgiou 2019), namely the standoff between the corporate community and the German government. This, I believe, is due to their neglect of the dimension of corporate power. Again, Frieden and Walter note a second major anomaly in the Eurozone crisis in relation to past balance-of-payments crises, namely that there was no debt restructuring for debtor member-states as part of the distribution of the adjustment effort. This was because European financial corporations mobilized to prevent such an outcome, pushing instead for fiscal liability mutualization. The choice between these two alternatives (debt restructuring vs fiscal union) was highly consequential in terms of institutional development, as the first would not have led to the institutional innovations mentioned above; indeed, the German government pushed for private sector involvement as a way to limit the liability of its taxpayers in future bailouts. My point here is that specific socio-economic actors (financial corporations) were the agents of the federation-building innovations and that they organized and acted transnationally in a way that does not fit intergovernmental accounts of the crisis.

### EU-US comparisons and comparative historical sociology

The crisis has also spawned a third type of literature, namely comparative federalism studies that typically try to draw lessons for the EU from the development of macroeconomic government institutions in the USA. This literature is part of a broader trend in European studies, namely the considerable growth in US-EU comparisons since the early 2000s (as documented in Tortola 2014). Tortola also notes the increasing conformity of this scholarship to mainstream political science as well as, crucially for my purposes, the fact that more than half the studies in his sample are of an explanatory type. In his view, “the most plausible explanation is that as the EU becomes more federal in nature and hence similar to the USA, causal work, which presents stricter requirements in terms of variable control, becomes easier” (Tortola 2014, 1347).

The strand of the comparative federalism literature that is most relevant for my purposes is work that looks at American political-economic and institutional history in an attempt to draw lessons for contemporary Europe (Bordo et al. 2011; Frieden 2016; Henning et al. 2012; Kirkegaard et al. 2018; Schelkle 2017; Steinbach 2015). Much of this work was commissioned by EU institutions or influential think-tanks—an interesting point in that it speaks to the frame through which European officials themselves think about the macroeconomic and institutional issues facing the EU. What all of these works have in common is a focus on the political dynamics that have fashioned the development of the American system of macroeconomic government. This places all of them in the broad category of the study of American political development, with the additional, albeit implicit most of the time, epistemological *parti pris* that EU development is of the same nature. This converges with my criticisms of the CPE and IPE literature.

However, the body of scholarship referenced above broadly suffers from two methodological shortcomings. The first is that there is no agreement on periodization. There is not even an attempt to theorize what is exactly comparable, why and how. The general impression one gets from reading the works is of a speculative excursion into American history in search for useful parallels with contemporary Europe. Some go back to the Hamiltonian Assumption, others look at the state bankruptcies of the late 1840s and others at the New Deal.

This observation also largely explains the second shortcoming: the literature offers no systematic testing of causal propositions that would provide a firm ground on which to develop the macro-causal analysis identified by Skocpol and Somers in their classic article (1980) as one of three basic modes of comparative historical analysis. As they put it, in this type of comparisons.

“the reason for juxtaposing case histories is to persuade the reader that a given, explicitly delineated hypothesis or theory can repeatedly demonstrate its fruitfulness – its ability convincingly to order the evidence – when applied to a series of relevant historical trajectories. … The point … is to assert a similarity among the cases – similarity, that is, in terms of the common applicability of the overall theoretical arguments” (176–177).

This is precisely the point of trying to draw lessons from American history for contemporary Europe. In its search for such lessons, the literature—probably unknowingly—makes the implicit assumption that the two cases have a profound and hidden, similarity that makes it possible to even draw lessons from one for the other. For if the two cases were fundamentally dissimilar in their underlying historical and political dynamics, there would be no point in trying to draw lessons.

In this paper, I propose to carry out a macro-causal analysis of the two cases along the methodological lines described by Skocpol and Somers. Crucially, the two shortcomings identified above can be addressed by adopting such an approach. By asking whether macroeconomic government institutional development in the two polities reflects a set of common drivers, i.e. by developing a common theoretical framework for understanding the two historical trajectories, one can begin developing a causal proposition that can help identify the specific historical sequences that are comparable. These must be sequences in which the common drivers are present and which yielded similar outcomes in terms of political development.

### Addressing the shortcomings: corporate reconstructions of federal polities compared

To do so, I proceed on the basis of three principal considerations.

First, I explicitly propose to study the evolution of the EU’s macroeconomic government institutions as part of the gradual building of a federal order—i.e. as European political development. In doing so, this paper is part of a broader project, sketched out in the past by Kathleen McNamara (2004 and 2010): developing a historical sociological approach to European integration based on comparisons with the US.

Second, I advance a specific proposal about the relevant period in American political history (namely, in broad brushes, the first half of the twentieth century) for the comparison with the EU.

Third, I offer a specific hypothesis about the social sources of the developments discussed, namely that the corporate reconstruction of the respective capitalisms is the root cause of both historical trajectories through the agency of large corporations and corporate elites.

The last two considerations go hand in hand, in the manner outlined at the end of the preceding sub-section. My argument is that the relevant comparison (in historical-sociological terms) for the Eurozone is with the first half of the twentieth century in American political development. This is because, firstly, this was when the contemporary system of federal macroeconomic government institutions gradually took shape—the Federal Reserve System was set up in 1913 and a gradual process leading to the peculiar shape of contemporary American fiscal federalism can be observed throughout the period, although the breaking point is the New Deal. Secondly, the underlying socio-economic reality that can analytically ground the comparison is the corporate reconstruction of American and European capitalisms. American corporate capitalism matured and asserted its political power in the first half of the twentieth century, whereas European integration can be analysed as both a necessary dimension and a consequence of the flourishing of corporate capitalism in Europe.^3^

This, then, is my basic causal proposition: both historical trajectories are idiosyncratic manifestations of a common macro-historical process, namely the advent of corporate capitalism and the subsequent reorganization of government institutions that this entails. The independent variable is the corporate reconstruction of capitalism, whereas the dependent variable is the development of (federal) institutions of macroeconomic government. The theoretical claim is that just as corporate capitalism requires large-scale markets to flourish and therefore pushes towards the large-scale centralization of economic activity, it also entails a similar reorganization of government institutions at the corresponding scale, including in the monetary and fiscal field. The intervening process here is corporate mobilization in favour of political development, i.e. the conscious deployment of the agency of corporate actors (leaders of large corporations and the organizations that they build collectively to further their interests and political aims) with the aim of restructuring government institutions so that they can fulfil new policy functions that are called for by the advent of corporate capitalism.

Let me spell this out in some more detail. The corporate mobilization in favour of new federal institutions does not occur in a political-economic and institutional void. The advent of corporate capitalism upends the previous pattern of economic activity and the political-economic institutions in which that pattern was embedded. This concerns both the geographic, legal, market and macroeconomic dimensions of the previous pattern. The upending of the previous pattern generates economy-wide tendencies to instability that are profoundly destabilizing and fundamentally at odds with the smooth functioning of the corporate economy. The resulting contradiction between the new pattern of economic activity embodied by the large corporation and the previous set of government and market institutions generates large-scale crises that do not simply impact the large corporations but also their labour forces and society more broadly. A public problem arises that calls for an institutional solution, which creates the political conditions for corporate mobilization to intervene as the mediating mechanism leading to political development.

In terms of macroeconomic policy, this translates into a quest for government institutions pursuing counter-cyclical stability policies that rein in the unchecked operation of market competition. Modern central banks have been tasked with pro-actively stabilizing the currency system and even with ensuring the smooth operation of financial markets and financial stability, whereas modern fiscal systems, as per the modern theory of public finance since Richard Musgrave’s seminal article (1939) outlining the three functions of fiscal policy (income redistribution, capital allocation and macroeconomic stabilization), have taken on both social welfare and macroeconomic stabilization tasks. My specific application of these insights to the two cases of interest here is that in the respective polities, this quest has also entailed creating new federal institutions and centralizing government powers at the federal level—thus leading to federal political development.

More specifically, my claim that the root cause of the respective historical trajectories is the corporate reconstruction of the respective capitalisms rests on a sequence comprising four steps. The first is looking at historiographical and theoretical debates about American political history and European integration for theoretical insights that point to the importance of the rise of corporate capitalism and large corporations in both cases. In this way, I show that existing, non-comparative, scholarship contains major elements for building a common, comparative theoretical framework. The second step is the microeconomic premises for the political developments that form the main focus of my comparative explanation. I look at the parallel processes of the rise of large corporations and the attendant politico-legal construction of single markets through the centralization of market regulation and economic law-making at the federal level. The microeconomic dimension logically and historically precedes the macroeconomic, in that corporate capitalism first has to take root before it generates the macroeconomic instability that needs to be addressed through new federal macroeconomic government institutions. The third step in the sequence is central banking and the need to restructure the monetary system in order to eliminate the monetary and financial instability that arises from the combination of the previous system and the rise of the corporate economy. This historically precedes the fourth step in the sequence, namely the restructuring and federalization of the fiscal system with the aim of creating federal economic shock absorbers tasked with macroeconomic stabilization at the scale of the single market.^4^

The rest of the paper uses the basic tools of comparative-historical analysis (Mahoney et al. 2003; Mahoney et al. 2015) to lay out the argument. For each case and in each section, I try to isolate from the broader historical context the causal link between the corporate reconstruction of capitalism and the institutional developments under examination. These developments were obviously influenced by many other factors, including other economic developments but also cultural trends, political regimes and the like. For example, the deeply rooted Jeffersonian aversion to federal authority and the associated commitment to states’ rights have been defining features of the history of central banking and federal fiscal policy (Zelizer 2003) in the USA. In the EU, the deep scepticism within German public opinion toward inflation and fiscal transfers created major political obstacles that have decisively shaped the process. However, for the purposes of the argument, these are set aside. Moreover, space considerations mean I can only provide a broad-brush outline of how the comparison works. As a result, I make extensive reference to historiography for the American case, whereas for the European case I present the gist of work published elsewhere. I devote a section to each case and each section is broken into four sub-sections that reflect the four-step sequence that structures the comparative explanation that I apply to the two cases: the first presents historiographical and theoretical debates about each case, the second sums-up the microeconomic process of corporate transformation and the parallel process of adjusting and federalizing regulatory infrastructure. The third sub-section looks at central banking and the fourth at fiscal developments. Finally, a concluding section takes stock.

## The American case

### The historiographical debate on the Progressive Era and the New Deal

The starting point when looking at the American case is the broad evolution of the historiographical debate^5^ on American history during the first decades of the twentieth century. This encompasses the Progressive Era (1896–1916) and then crucially the New Deal.

The first school of thought that developed in the 1950s and 1960s—around the works of progressive historians like Arthur Schlesinger Jr. and William Leuchtenburg—was the liberal interpretation, in which the rise of “big government” and federal regulation was a manifestation of the rising power of the common people against business interests, the victory of liberalism over conservatism and state control of markets over laissez-faire liberalism.

This angelic interpretation was quickly countered by a number of other interpretations, which converged around the idea that the profound transformations were at least congenial to the needs of the large corporations that had sprung up at the turn of the century, if not their very creation. The revisionist school coalesced around the work of William Appleman Williams (1961), Gabriel Kolko (1963) and James Weinstein (1968) and argued that the rise of “big government” was driven by the large corporations’ quest for taming market competition, reconstructing industrial relations on a cooperative footing and stabilizing profits.

Historian Robert H. Wiebe’s seminal *The Search for Order* (1967) provided a bridge between these revisionists and another school of thought that emerged in the 1970s, namely the organizational synthesis interpretation (the seminal statements are Galambos 1970 and 1983). This interpretation built on leading business historian Alfred Chandler’s pioneering work on the second industrial revolution leading to the rise of large corporations (1977, 1990) and bureaucratic hierarchies as the organizing principles in the new age of corporate capitalism that gradually came into being from the late nineteenth century onwards. Technological imperatives led to economic reorganization around the large corporation, whose leaders played a leading role in establishing big government as a way of uniformly organizing markets at the national scale, taming destructive price competition, stabilizing labour markets and organizing the supply of various kinds of professional experts necessary to run complex bureaucracies.

Finally, in this connection, one would have to mention the sociological tradition on the power elite, pioneered by C. Wright Mills (1956) and then further developed by a range of authors, in particular G. William Domhoff (2007 and 2017) but also others such Mark Mizruchi (1992, 2013), Beth Mintz et al. (1985) and Michael Useem (1984). These authors have different assessments about the precise extent and shape of corporate power, but their unifying theme is how corporate capitalism ushered in a power structure at the centre of which one finds the leaders of America’s largest corporations. This literature extensively documented how the corporate elite organizes in order to wield power, through interlocking directorates, the policy-planning network, campaign finance, the revolving door with federal bureaucracies, corporate political action committees and the like.

These schools converge around the concept of “corporate liberalism”: the idea that corporations and their leaders were the key drivers behind the “liberal” reforms of the Progressive Era and the New Deal that introduced a break with nineteenth century laissez-faire. The unifying concept for understanding these related transformations in economy, society and polity is provided by historian Martin J. Sklar’s analysis (1988) of antitrust policy: *The Corporate Reconstruction of American Capitalism*. In a historiographical essay (1991) on periodization and American political development, Sklar sums up the problem succinctly (emphasis my own):

“In general, periodization with reference to the emergence of the corporate reorganization of American capitalism assumes broad social change engendered by the extended conflict between *two major forms of capitalist property relations* and their corresponding modes of consciousness, or, between *two historical stages of capitalist society*, the *proprietary-competitive market stage* and the *corporate-administered market stage*, the one receding before, but having generated and therefore leaving its indelible marks upon, the ascendancy of the other.” (208).

This, then, is the deep unity that links together developments in institutional development in the USA in the first decades of the twentieth century. The next section outlines the broad pattern of the rise of American corporate capitalism and a national market as well as the regulatory developments associated with it.

### The search for microeconomic order and the rise of the federal regulatory state

Alfred Chandler’s work is the starting point for any account of the rise of corporate capitalism in the USA. Chandler showed that before the 1880s, the only large-scale business organizations were the railroads. In his account, the emergence of the large corporation was fundamentally driven by the organizational requirements of the technological innovations of the second industrial revolution. These required substantial fixed investments and much greater production volumes if they were to be profitably exploited. The much greater technical sophistication of mass production also entailed a much greater division of labour within any given firm and this led to functional specialization and bureaucratization of managerial tasks, leading to the functional separation between ownership and management.

The key turning point in this process was the great merger wave of 1895–1904 (Lamoreaux 1985). As Sklar has shown, this was spurred by the Sherman anti-trust act of 1890, whose avowed objective was to block cartelization of American industry and the trend towards monopolization. Instead, the Act pushed business leaders towards the alternative path of consolidation and thus sped up the process of corporate transformation of productive structures.

This microeconomic process drove the trend towards economic nationalization. Michelle Egan, in her comparison with the construction of the European single market (2008, 2015), shows how the rise of large corporations straddling the geographic boundaries of the states drove a process of convergence in prices and integrated product markets. This process was accompanied by financial integration; economic historian Lance Davis (1965) showed how during the period 1870–1914, interregional interest rate differentials converged and corresponding capital flows grew, thus leading to the rise of “a national capital market”. Similarly, historian Richard Sylla analysed (1969) how the banking and monetary system of the time (the National Banking System) enabled the supply of growing volumes of funds to the US main financial centres where they were used to finance railroad and industrial development.

The trend towards single product and financial markets also had clear legal and regulatory implications. Once again, corporate agency was key in this regard, as there developed, during the last quarter of the nineteenth century “a noticeable tendency for big business to seek federal legislation in order to avoid the problems of adjusting to multiple state laws.” (Egan 2008, 263). Gabriel Kolko’s study of the federal regulation of the railroads (1965) and the active role played by railroad executives in seeking and defining the substance of such legislation is the typical example of this process.

The judicial system was similarly drawn into the fray. “Leaders of national business deemed the federal courts friendliest to their interests” (Freyer 1979, 345) as opposed to state courts still under the sway of localistic interests. These leaders therefore made extensive use of the constitutional right to move cases from state to federal district courts. The federal judicial system responded accordingly and was “instrumental in overcoming local resistance to national business” (344) by helping to develop through jurisprudence a national commercial law that decreased the heterogeneity and discriminatory character of state commercial regulation and promoted interstate business and legal certainty and uniformity. More broadly, legal historian Harry Scheiber argues (1975) that the decentralized legal system of “rivalistic state mercantilism” of the pre-Civil War era gradually gave way in the decades to 1910 to a much more centralized system in which federal legislation and courts were used to put together a uniform national economic system dominated by the large corporations.

This process went hand in hand with the rise of the national administrative state, a process described in American Political Development’s foundational work, Stephen Skowronek’s *Building a New American State* (1982). Skowronek argued that during the period 1877–1920, an epochal transformation took place where a polity based on “a state of courts and parties” that represented isolated local communities gave way to a national administrative state under the weight of the forces unleashed by “industrialism”.

“[T]he pivotal turn away from a state organization that presumed the absence of extensive institutional controls at the national level toward a state organized around national administrative capacities. [Their expansion] around the turn of the century was a response to industrialism. The construction of a central bureaucratic apparatus was championed as the best way to maintain order during this period of upheaval in economic, social and international affairs.”

What was the link between corporate capitalism and the national administrative state? As Chandler has shown, the corporate transformation of the economy came with a profoundly altered competitive dynamic among firms. Whereas in the proprietary-market stage, competition was waged mainly through price and wage cutting, in the new corporate stage, firms competed mostly on market share and cost cutting through efficiency-enhancing investments. This meant that large corporations needed to alter the legal and regulatory underpinnings of market competition to tame competition from new entrants, collectively control supply so as to stabilize market shares and eliminate price wars. This was the microeconomic variant of Wiebe’s “search for order”. The federal regulatory agencies that sprang up from 1887 onwards, with the creation of the Interstate Commerce Commission to regulate interstate railroads, precisely enjoyed the support of the corporate community because they could set floors to prices and raise entry barriers to markets, thus stabilizing competition. The trend culminated with the NIRA in 1933, a legislative act largely inspired by the Swope plan of 1931 authored by General Electric’s president Gerard Swope, a leading corporate liberal (McQuaid 1978, 352–354 and 2003, 171–174) who would play a key role during the New Deal. Swope’s plan called for sectoral associations empowered to bargain on prices, production quotas and wages. The NIRA created instead a federal agency (the National Recovery Administration) that would enforce federal sectoral regulation with the same aim. In the meantime, corporate leaders had come to appreciate the compulsory dimension entailed in federal enforcement because they grew disenchanted with the voluntary basis of plans for sectoral associations. The NRA was struck down by the Supreme Court in 1935, but its basic aims were furthered by federal legislation on hours and wages and social security.

### From the National Banking System to the Federal Reserve System

The same logic of taming unruly markets and bringing order to the American banking and monetary system presided over the corporate push for the creation of a federal central banking system.

The banking and monetary context within which rose the large corporations was the National Banking System established during the Civil War through the 1863 and 1864 National Banking Acts. The system had a number of crucial features, whose combination with the rise of the corporate economy would create systemic instability in the late nineteenth and early twentieth century (for a summary, see Rousseau 2018). In particular, the system gave rise to regular banking panics that grew in frequency and intensity from 1890 onwards. It was in response to this structural instability that corporate reformers gradually converged towards and mobilized in support of a central bank that would centralize bank reserves, counter-cyclically manage the currency and act as a lender-of-last-resort in order to stabilize financial conditions (Livingston 1986 and Lowenstein 2015).

The National Banking System had been born out of an attempt to create a permanent and stable market for federal government debt to finance the war effort. Accordingly, nationally chartered banks were allowed to issue banknotes backed by their holdings of federal government bonds, whereas state chartered banks were all but prohibited from issuing banknotes by a 1866 tax on issuance. This was the main reason for a crucial defect of the system, which contemporaries used to refer to as the “inelasticity” of the currency, namely the fact that the money supply could not be centrally manipulated for counter-cyclical purposes. This was compounded by the seasonal variations in the demand for credit that resulted from the operation of the agricultural economy and the marketing of crops. Agricultural demand for credit would peak in the spring and autumn, thus creating seasonal peaks in interest rates. Finally, as the USA grew richer and bank deposits more plentiful, the American money market was organized in an “inverted pyramid” structure, whereby country banks would park their reserves with banks in reserve cities and the latter would lend these on to national banks in the three central reserve cities of Chicago, New York City and Saint Louis. Seasonal peaks in the demand for credit and money would therefore regularly create conditions of financial stringency in New York, the nation’s financial capital, as the pyramid of bank reserves would unwind to meet the increased demand coming from the countryside.

A final link in the chain that would become a big source of financial instability was the link between these “bankers’ balances” and the New York stock market via the operation of the call loan market (Calomiris 2013, 171–172; Livingston 1986, 137–145). The call loan market was key because it crystallized the interpenetration of the money and capital markets. When New York City banks were awash with reserves, they would lend them on call to stock market operators who would use them to invest in securities. This would create inflationary dynamics in securities prices that made busts extremely likely when the inverted pyramid unwound in the spring and autumn. This became a major concern after the securities market was given a major boost by the great merger wave and the securities of industrial corporations became a major feature on the stock market alongside those of the railroads (O’Sullivan 2016). The first decade of the twentieth century was therefore dominated by concerns about the call loan market destabilizing the stock market.

These features were compounded by two institutional dimensions of the system. The first was the prevalence of a “unit banking” structure and the lack of branch banking (Bordo et al. 2013, 61–63). A legacy of the Jeffersonian and Jacksonian aversion to concentrated financial power was the variety of legal rules that restricted the development of banks with multiple branches, in particular interstate branching. “Unit banking” meant that the geographical and sectoral diversification of bank assets was weak, which heightened banks’ exposure to economic shocks and fuelled the competitive hoarding of reserves during panics. The second dimension was that this decentralized structure lacked an institutionalized lender-of-last-resort who would take responsibility for restoring money market stability during panics.

Livingston provides a very detailed account of corporate mobilization in support of the reform of the banking and monetary system and the gradual forging of a consensus around the need for a central bank.^6^ He shows that from the very beginning of the corporate movement for reform (which he dates to the 1897 Indianapolis Monetary Convention), the major preoccupation of corporate elites was to create centralized control over the market. As put in the 1906 report of the currency committee of the New York Chamber of Commerce, which was the first formulation by a body representative of corporate interests of the proposal for a central bank: “there is no centralization of financial responsibility, so that in times of dangerous over-expansion no united effort can be made to impose a check which will prevent reaction and depression” (Livingston 1986, 161). In the movement’s first years (1897–1906), the main demand was for reforms allowing for the development of branch banking and providing for an alternative basis on which to issue currency (banknotes) so as to both centralize control over reserves and render the money supply more “elastic”. But opposition to branch banking was so entrenched in the countryside and Congress that Livingston claims that by 1902 corporate reformers were convinced of its political infeasibility. For a brief period until 1906, they toyed with the notion of having the Treasury act as a crude central bank—as indeed Treasury secretaries Lyman Gage (1897–1902) and Leslie Shaw (1902–1907) attempted to do, by depositing federal revenues with New York banks at times of stress. However, the funds available to the Treasury were insufficient for it to play that role and this helped bring round the corporate community to the conclusion that only radical reform, i.e. the setting up of a central bank, would work (Livingston 1986, 155–158).

This placed centre-stage Paul Warburg, the Kuhn, Loeb investment banker who is widely credited in the historical literature on the origins of the Federal Reserve System as being the main intellectual instigator of the provisions of the Federal Reserve Act of 1913. Warburg had been privately calling for a central bank for a few years. His view was that broadening the basis for banknote issuance was dangerous, because this would allow small country banks to extend credit far too easily and allow “marginal” producers to continue operating, thus leading to over-production and price wars—precisely the problems that the “search for microeconomic order” was trying to deal with. He further believed that the practice of loaning surplus funds against securities as collateral on the call loan market was a major source of financial instability. His proposed solution was to reorganize the American money market by replacing the call loan market with a discount market for bankers’ acceptances, organized around a central bank whose main function would be to use the rediscounting of such paper as a way of counter-cyclically managing the money supply (O’Sullivan 2016, chapter 6). In particular, in periods of stringency or panic, banks would find it possible to easily rediscount such assets with the central bank, instead of scrambling for cash and cutting back on loan creation.

After the panic of 1907, during which J.P. Morgan played the role of a central bank by assembling New York bankers and coordinating a rescue using government and private funds, the movement for reform gained momentum. A National Monetary Commission was created in 1908, in which Senator Nelson Aldrich, the corporations’ most trusted political ally in Congress, played the leading role. Warburg and Aldrich worked together, secretly coming up with the Aldrich plan during the now famous Jekyll Island meeting in late 1910. Warburg then instigated the creation of the National Citizens’ League for the Promotion of a Sound Banking System, the corporate campaign group in favour of the Aldrich Plan.

Much has been written about the Congressional drama that ensued and about the differences between the Aldrich Plan and the Federal Reserve Act adopted by a Democratic Congress and President. However, the basic functions of the Federal Reserve System were identical to those envisaged by Warburg and “most of those involved felt that the goals of the Aldrich Plan and of the Democrats’ draft legislation … were more or less the same” (Livingston 1986, 216).

One dimension of the 1913 FRA that diverged from Warburg’s plans was the level of centralization of power. Under the influence of Democratic Representative Carter Glass and Senator Robert Owen, who both harboured a Jeffersonian suspicion of concentrated financial and federal power, the FRA provided for substantial decentralization with the twelve Federal Reserve Banks retaining the power to pursue their own discounting and open market policies. Bordo et al. (2013, 86–88) blame the failure of the Federal Reserve to play effectively its role as a lender of last resort in the early 1930s on this decentralization of power. This defect was addressed through the 1933 and 1935 Banking Acts that substantially centralized power at the Federal Reserve Board and the Federal Open Market Committee.

A second defect remained as well: the FRA only obligated nationally chartered banks to join the System, not state chartered ones and the legal obstacles to interstate branching remained intact for decades. As a result, a big swath of the American banking industry remained both highly fragmented and outside the purview of the System. This hampered the System’s ability to pursue counter-cyclical policies and prevent banking failures at times of economic stress—a limitation that contributed to the failures of the Federal Reserve during the 1930s.

### Reconstructing the fiscal system: the rise of big government and fiscal federalism

**Fig. 1:**
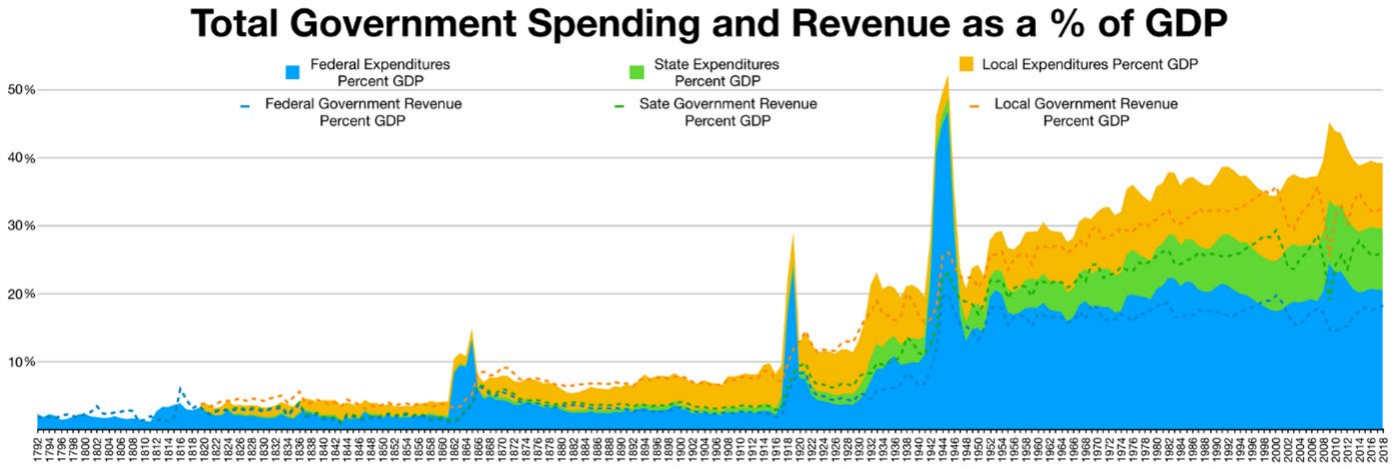
Source: Wikipedia article “Government spending in the United States” https://en.wikipedia.org/wiki/Government_spending_in_the_United_States

The story of the rise of contemporary American fiscal institutions is that of two trends coming together during the 1930s to produce what John Joseph Wallis has termed the “birth of the old federalism” (1984) or the third American system of government finance (2000). These two trends (depicted in Fig. 1) are, on the one hand, the steep growth of government spending (big government) and, on the other, the growth of the share of federal spending in the total (fiscal federalism).

This fiscal regime has three distinctive features. On the revenue side, it relies on income taxation. On the spending side, the federal government has both assured the bulk of the growth in total outlays and become the main government spender. Finally, the system regularly records federal budget deficits that are largely due to the entrenchment of a peculiar Keynesian conception of fiscal policy based on a preference for automatic stabilizers and tax cuts over greater discretionary spending.

The New Deal period is, therefore, crucial for understanding the contemporary American fiscal regime. It was during those years that the federal social welfare programs responsible for the secular growth in total outlays were legislated, that a Keynesian conception of a counter-cyclical fiscal policy replaced the previous mantra of balanced budgets and that income taxation was stepped up to provide government with the necessary revenues. The historiography of the period offers abundant evidence for the key role played by corporations and their leaders in shaping these developments. For these leaders, these developments were tools for stabilizing the corporate economy—they were thus guided by a “search for macroeconomic order” in a spirit similar to the “search for microeconomic order” described above.

In general terms, the broad historical explanation for the growth of big government is that it was a response to the second industrial revolution that ushered in corporate capitalism.^7^

“Increasing specialization and division of labor … caused acceleration in the rate of government growth. The initiating force was the wedding of science and technology in the nineteenth century. … The consequent capital-intensive technology entailed large-scale continuous production and distribution and set in motion a set of fundamental changes in every aspect of society. … The consequent restructuring … led to increased demands on government both to assume functions previously performed by private organizations and to respond to the pressures of the increased number of special interest groups.” (North 1985, 388).

The corporate economy created vast needs for public goods such as education, health, infrastructure spending and social insurance. In his book on the co-evolution of the “twin revolutions” of corporate capitalism and the activist state, R. Rudy Higgens-Evenson (2003) has documented how the trend towards the growing provision of these public goods began at the local and state level in the last quarter of the nineteenth century and how it stimulated a growing involvement by corporate leaders in the administration of public policies and in budget management, resulting in the adoption of business methods by government bureaucracies. Higgens-Evenson also shows how this trend lies behind another important change in the fiscal regime, namely the transition from property to income taxation. Property taxation had made intuitive sense so long as the American economy was primarily agricultural and the main form of wealth was land and real estate. But this was no longer enough to generate the revenue needed for investment in public goods provision and so local and state governments began looking for alternative revenue sources starting in the 1870s. Corporate income tax was their solution and corporations were sympathetic to it due to the inconsistent application of property taxation. But corporate leaders insisted that if they were to pay for such public goods provision, they needed to be able to exert substantial influence over taxation and spending. Hence their growing involvement in government policy-making and their insistence on applying business management methods to public administration. “By 1929, the rise of the corporate state had fundamentally changed the relationship between government and business. Business officials had gone from bribing and blackmailing state legislators to helping them run government on a paying basis.” (Higgens-Evenson 2003, 9).

The novelty of the New Deal was that the federal government would now massively take up these expenditures. The rise of civilian federal spending starting in the 1930s can be broken down into two sub-groups: infrastructural and other “internal improvement” spending, used during the 1930s to counter-cyclically fight the Depression and new federally funded social insurance schemes legislated through the 1935 Social Security Act.

The most consequential innovation in terms of the long-term growth of federal outlays was the adoption of the 1935 Social Security Act, which instituted federal old-age and unemployment insurance. The literature on the SSA is vast and is structured by a long-standing controversy about whether the SSA was legislated against business or at its instigation. The scholarship pursuing the latter explanation (Berkowitz et al. 1988; Swenson 2002; Domhoff et al. 2011) is particularly robust because it makes extensive use of archival material and because it is based on a sound understanding of the functional logic that animated corporate supporters of a federal welfare state. Swenson has demonstrated that for large corporations, federal social insurance was a means of stabilizing, generalizing and perpetuating the wage-setting system based on collective bargaining, efficiency wages and private welfare schemes they had gradually instituted over the preceding decades. Mandatory federal schemes would raise the incompressible wage costs facing all firms in any given industry to the levels incurred by the large corporations. Moreover, they would even out the geographical discrepancies that had built up due to the many state-level schemes adopted since the mid-1910s (Swenson 2002, 201–207 for such examples). This would raise additional entry barriers and prevent “chiselers” and “marginal producers” from destabilizing entire industries through price wars based on wage cutting. These large corporations were also interested in old-age insurance as “a way to replace superannuated workers with more productive younger workers” (Domhoff et al. 2011, 143). Finally, another function for these federal schemes that corporate leaders, especially those in consumer industries and mass retailing, perceived was the stabilizing effect on aggregate purchasing power they would create by setting a floor below which workers’ incomes could not drop.

The legislation itself was largely drafted by experts from Industrial Relations Counselors Inc., a consultancy funded by corporate money that was also to provide many of the administrators who would staff the federal agencies set up to administer the programs. As a result, “the specific principles embodied in … the SSA came from corporate experience with private insurance plans” (Domhoff et al. 2011, 143). Indeed, the Swope plan of 1931 included provisions for legally mandatory unemployment, old age, life insurance and disability programs that would be decided and administered by national sectoral bodies (McQuaid 1978, 353–354). Roosevelt asked Swope himself in March 1934 to submit detailed proposals for legislation; GE’s president also became the most prominent corporate member of the Citizens’ Advisory Council on Economic Security, the body attached to the cabinet-level Committee on Economic Security that was responsible for drafting the legislation. Swope and the other corporate supporters of the generalization of insurance schemes were converted to the idea of legally mandatory, federal, publicly administered insurance schemes because they understood that these would “force upon competitors the standards that progressive employers were struggling to adopt” (Swenson 2002, 203–204). Once again, direct federal governance instead of voluntary sectoral associations was preferred for their compulsory dimension.

The Swope plan is a good transition to the second distinctive feature of the new fiscal regime, counter-cyclical fiscal activism. The plan included provisions for a vast federal public works program (McQuaid 2003, 171–173). In doing so, it took up an idea that had begun spreading within the corporate community in the 1920s and extensively advocated for in 1930–32. In the initial stage of the Depression, from 1929 to mid-1931, the Hoover administration had pursued an expansionary fiscal stance (Stein 1969, chapter 2 and Brownlee 2016, 117). After an interlude of fiscal tightening in mid-1931 to 1933, the thrust towards expanded public works spending regained momentum under Roosevelt with the support of the corporate progressives in the Business Advisory Council.

However, it was not until Roosevelt’s April 1938 fiscal turn, following the 1937 recession, that the orthodoxy of balancing the budget as the basic guideline for fiscal policy was profoundly challenged, within both the administration and the corporate community. In both cases, a minority within the corporate community was instrumental in bringing about a change of views (Collins 1981; Stein 1969, chapters 6 and 8). The key individuals in this respect were Beardsley Ruml and Paul Hoffman, who would play a leading role in the Committee for Economic Development, the corporate lobby that dominated post-war economic policy (Mizruchi 2013, chapter 3; Domhoff 2013; Stein 1969, chapter 9^8^). Collins and Stein document the parallel conversion of the corporate community and successive administrations from Roosevelt to Kennedy to a conservative version of Keynesianism emphasizing deficit spending to fight recessions while balancing the budget over the business cycle and in-built counter-cyclicality based on automatic stabilizers. This version of Keynesianism also contained a strong preference for generating budget deficits through tax cuts rather than increases in discretionary spending.

Ruml and the corporate leaders involved in the BAC and then the CED were also key supporters of the transformation of federal taxation, the third distinctive feature of the new regime. During the “second New Deal” in 1935–38, Roosevelt toyed with “soak-the-rich” income taxation, in particular the undistributed profits tax. The BAC and key corporate supporters of the New Deal opposed this tax program and by 1939 Roosevelt abandoned it (Leff 1985 and Brownlee 2016, chapter 5). Instead, the 1942 Revenue Act marked the fundamental transformation of the federal taxation regime through the adoption of “what became the core of [the] new tax regime—a personal income tax that was both broadly based and progressive” (Brownlee 1996, 91–93). The broadening of the base to include middle-class households had been a major corporate demand and one that embodied the abandonment of the initial redistributive logic associated with the early progressive case for an income tax (King 1983). As summed up by the most prominent historian of US federal taxation, in the new system “mass taxation replaced class taxation”, as the number of individual taxpayers grew from 3.9 to 42.6 million between 1939 and 1945. The Federal income tax in 1940 was responsible for 16% of all fiscal revenues, whereas by 1950 that figure was 51% (Brownlee 1996, 91–93). According to Brownlee, the wartime transformation of federal taxation and the parallel corporate conversion to budget deficits marked the end of the conflict over the fiscal regime between business and the progressive movement and its final recasting along corporate liberal lines.

## The European case

### The theoretical debate on the sources of European integration

The theoretical debate about the sources of European integration has developed largely within the sub-field of International Relations and builds on the conceptual tools of the discipline, as the initial framework within which scholars approached the issue was regional integration theory (Haas 1961). If anything, as scholarly debate unfolded, the idea that European integration was fundamentally about interaction between sovereign states became more entrenched, as the main rival to the original neo-functionalist approach that emerged over the years was liberal intergovernmentalism (Moravcsik 1998), a version of neorealist intergovernmentalism based on liberal theories of national preference formation, interstate bargaining and international organizations.

Whatever their differences, these theories agree that the independent variable and driver of the process is the rise and deepening of economic interdependence. For neo-functionalists, this generates functional pressures for breaking down barriers to international economic activity that are mainly given expression by transnational actors (MNEs, trade unions and the like) and the initiatives of the supranational institutions, mainly the Commission. Over time, the process is expected to lead to greater accumulation of power at the European level. Intergovernmentalists agree that such functional pressures drive developments, but they argue that they manifest themselves primarily through the action of the member-state governments, towards which economic actors maintain their primary political allegiance. The supranational institutions are no more than secretariats tasked by the principals (the member-states) with policing the contracts (the treaties and secondary EU legislation) that underpin the breaking down of barriers to pan-European economic activity. Intergovernmentalists are therefore the staunchest proponents of a theoretical model entirely informed by IR, whereas neo-functionalists uneasily straddle the theoretical divide between IR and political development, starting with concepts from IR but predicting that somehow the process will lead to a European federation.

These debates are obviously full of insights and their shared focus on economic interdependence is a major theoretical achievement. My starting point, then, is to question their understanding of interdependence. In their perspective, interdependence is a macroeconomic phenomenon that somehow “just happens” and that can be measured by the growing volume of intra-EU trade, FDI and financial flows. This is inadequate because it does not ask the all-important question of why deepening economic interdependence happens and therefore misses the microeconomic drivers of the process.

These drivers lie precisely with the corporate transformation of capitalism in Europe and are familiar to business historians of the corporate form and to IPE scholars concerned with the role of large corporations, scale economies and cross-border value chains in the setting up of regional trading blocs (Chase 2005 is the key work in this connection). In a comparative historical essay on the rise of large corporations across the advanced capitalist world, Chandler and his co-authors (1997) argued that the technological innovations responsible for the rise of corporate capitalism occurred simultaneously in the USA and Europe during the last quarter of the nineteenth century. However, the USA took the lead in the process of corporate transformation because in Europe the political-economic fragmentation that resulted from the multiplicity of powerful nation states acted as an obstacle that slowed down that transformation. This was mainly due to the persistent fragmentation of the European market into distinct national compartments, protected by tariffs and other barriers to trade. Consequently, European integration has been a key enabling dimension but also a consequence of the trend towards the corporate reconstruction of European capitalism. Jean-Christophe Defraigne’s (2004) economic history of European integration precisely provides a narrative structured around the rise and development strategies of large corporations in Europe and links this process to developments in integration. Chase’s IPE account of integration schemes in the 1930s and the push to complete the single market in the 1980s explicitly links these developments to the imperative of enabling firms to operate at their minimum efficient scale and therefore to the process of corporate transformation.

Seen in this light, European integration as a historical process shares more common features with the process of corporate transformation and the associated developments of economic nationalization and regulatory federalization in the USA summed up earlier than with processes of international economic liberalization that are the bread and butter of IPE. The parallel that I draw between these two historical processes is that the corporate reconstruction of capitalism is the common independent variable shaping both. Just as the rise of corporate capitalism entailed the profound remaking of money and banking as well as the fiscal regime in the USA, so it can be hypothesized that a similar process has been underway in the European Union.

### The rise of a pan-European corporate capitalism and federal regulation

The founders of the EU, notably Jean Monnet, as well as their American backers in the American federal government and the Economic Cooperation Administration (whose first Administrator was CED founder Paul Hoffman) had a very acute understanding of the link between integration and corporate capitalism. Their basic design was to foster the corporate reconstruction of European capitalism by moving towards the creation of a single European market of a scale similar to the American domestic market. Sectoral consolidation could take place in such a market in a way that would allow large pan-European corporations to develop and reap the economies of scale and scope that had ensured American prosperity and power.

In practice, this process unfolded in two stages. The first stage lasted until the mid-1970s. During this time, European integration was essentially a matter of removing quantitative and tariff barriers to trade and spurring the growth of trade flows among member-states. Parallel to this, sectoral consolidation in a few large corporations took place within each member-state, in many cases (in particular France and Italy) with the active involvement of state bureaucracies (Vernon 1974), in what became known as the strategy of building national champions. What little cross-border investment there was, was of a market- not efficiency-seeking nature and large corporations did not yet begin to integrate their production and distribution networks across the borders of the member-states—value chains remained organized nationally. The European market thus remained a collection of functionally distinct national economies, not an organic whole.

By the late 1960s, these parallel processes reached their limits. The customs union was completed in 1968 while the limits of the national champions strategy were becoming apparent in the more technologically advanced industries, where the lack of scale meant Europe remained far behind the USA and even Japan (Servan-Schreiber 1968). It was precisely at this point that the notion of Europeanizing the strategy of voluntaristically building large corporations arose and the launching of the Airbus consortium in 1970 was the first concrete manifestation of this new stage in industrial development.

The national champions began expanding throughout the European market from the 1970s onwards and restructuring their value chains to integrate them across member-state borders (Franko 1976). This trend really took off from the early 1980s onwards and two merger waves—one in the late 1980s and another in the late 1990s—marked its culmination. By the early 2000s, the European market had indeed developed into a single market dominated by pan-European industrial corporations operating integrated production and distribution networks (Rugman 2005; Rugman et al. 2005; Véron 2006).

This microeconomic development spurred the rise of a specifically European corporate elite. Studies of interlocking directorates (e.g. Heemskerk 2013) conclude that the ties linking together Europe’s largest corporations had grown substantially by the 2000s. Moreover, from the early 1980s a set of highly influential pan-European corporate lobbies began taking shape. The most important of these has been the European Round Table of Industrialists (van Apeldoorn 2002), founded in 1983 at the instigation of the Commission’s then vice-president, Etienne Davignon, the key figure in the expanding nexus between European institutions and corporate boardrooms.

As Europe’s corporations began Europeanizing, a parallel development of regulatory federalization took place. Although tariff barriers had already been struck down, a host of non-tariff barriers persisted, largely due to the multiplicity of member-state regulatory and procurement policies. Accordingly, the key to completing the single market was to move to uniform standards adopted at the European level. This was one of the key demands of the ERT when it was set up in 1983 and all scholarly accounts of the 1986 Single European Act—which created the politico-legal conditions for uniform European regulation—attribute to the ERT’s mobilization a determining role in bringing it about (Chase 2005, chapter 5). The SEA enabled the adoption of some 300 directives (EU framework legislation) that standardized regulation on all sorts of product and services markets by 1992. On this basis, a swath of EU regulatory agencies has sprung up over the last three decades.^9^ A parallel development was the federalization of competition policy. This included legislation prohibiting discrimination based on member-state domiciliation in public procurement and member-state aid to individual firms as well as granting the Commission powers to police cartels, abuses of dominant market positions as well as mergers and acquisitions.

### From the European Monetary System to the Eurozone

The Europeanization of former national champions was also the key driver of the push to create a single currency. Like their American peers some nine decades earlier, Europe’s corporate leaders were primarily interested in establishing conditions of monetary stability without fragmenting the single market of which they had become the backbone.

As these corporations began restructuring their production and distribution networks across Europe, the share of their total assets and revenue generated within the European market but beyond the confines of their home member state expanded significantly. Their Europeanization also generated increasing cross-border financial flows within the EU and this led large corporations to demand an end to the capital controls that were still in place in most member states in the early 1980s. Although the Treaty of Rome had laid down the four freedoms (free movement of goods, services, labour and capital), its provisions for capital included safeguards that in practice allowed full discretion to member-states on the matter. Such controls were used by those member-states that had chronic deficits in their current accounts (in particular, France and Italy) to protect their national currencies from destabilizing runs on the currency markets but also as a means of preserving domestic savings for domestic investment. Consequently, as former national champions began Europeanizing, the pressure to lift capital controls grew much greater, as these corporations began prioritizing the ease of moving funds across member state borders over privileged access to the savings generated in their home member states.

However, the growth in cross-border flows and the parallel removal of capital controls created conditions of increased monetary instability. This happened for two reasons. Firstly, deeper financial integration meant deepening macroeconomic imbalances within the European market between a bloc of surplus member states around Germany and a bloc of deficit member states around France. This was the traditional structure of imbalances going back to 1953, when Germany generated its first post-war current account surplus. However, deeper capital markets now meant that the imbalances could be financed more cheaply for longer and thus grow in relative magnitude before triggering capital flight (James 2012, 1–28). Secondly, the removal of capital controls eliminated an effective tool for slowing down capital flight. It therefore heightened the pressure on deficit member states to align their macroeconomic performance on that of Germany, which had become the pivot of the system of fixed but adjustable exchange rates created in 1978 (the European Monetary System) and thus the European benchmark for financial investors.

The EMS grew increasingly unstable over the years from 1978 to 1992, despite the fact that a process of macroeconomic convergence did actually take place during the first half of the 1980s (Andrews 1994). Its inherent instability was highlighted in the late 1980s, when the dollar’s renewed weakness post-1985 sent a wave of capital to Europe that led to diverging pressures on various national currencies and unwarranted monetary tightening in France and Italy. The monetary crisis of 1992–1993 that in essence destroyed the EMS was a perfect illustration of the system’s deficiencies.

As the EMS’s dysfunctionality became increasingly obvious, corporate leaders concluded that the EMS had to be replaced by a single currency run by a federal central bank so as to eliminate the instability by simply eliminating national currencies and the attendant currency risk altogether. As Jeffry Frieden has amply demonstrated (1996 and 2002), the Europeanization of national champions gradually led them to reverse their policy preferences to favour monetary stability over domestic policy autonomy since a growing share of their activities became exposed to currency risk. Consequently, “[h]igher levels of cross-border trade and investment increase the size and strength of domestic groups interested in predictable exchange rates. Firms with international ties support a reduction of currency fluctuations. These effects are especially important to banks and corporations with investments throughout the EU” (1996, 202).

The empirical record of corporate mobilization in favour of the single currency fully backs this interpretation. A campaigning organization was set up in 1987, called the Association for the Monetary Union of Europe (Davignon was its president from 1991 until it disbanded in 2002) and it very quickly assembled a membership that was sectorally and geographically representative of corporate Europe. Two of its managing directors summed up the rationale of its members: “Practical men in Europe were confronted with high costs due to monetary instability. Given the growing degree of European market integration they favoured exchange rate stability” (Collignon et al. 2003, 50). This was confirmed to me in interviews with Davignon himself (Brussels, 12 January 2018) and Yves-Thibault de Silguy (Paris, 24 November 2017), the commissioner responsible for the euro in 1995–1999. De Silguy was emphatic about the crucial role played by AMUE in bringing the project to fruition, in particular in Germany where AMUE launched a vast campaign that swayed public opinion. He also emphasized the extremely close coordination between the Commission and AMUE as well as the key role played by corporate leaders in influencing their respective national political leaders.

The deal struck in Maastricht in 1991 was thus very much in line with corporate preferences. Crucially, the corporate mobilization overcame the greatest obstacle on the road to a single currency, namely opposition in German public opinion and by the Bundesbank. German public opinion was highly sceptical of the project due to an entrenched aversion to inflation and a fear of unwillingly sliding into a fiscal union entailing transfers from richer to poorer member states. AMUE therefore supported the safeguards demanded by the German government in a bid to assuage its domestic constituents: a mandate focused on the pursuit of price stability (article 127.1 TFEU) as well as treaty clauses prohibiting the mutualization of fiscal liability (article 125 TFEU) and monetization of public debts (article 123 TFEU).

Although corporate leaders had an intellectual understanding of how introducing a single currency would in all likelihood create a need to move towards fiscal union as well, the immediate problem they were facing was monetary instability. Consequently, their primary concern at the time was to secure monetary union and so they decided against insisting on more institutional innovations out of a fear of creating additional political obstacles on the road to a single currency. As one of the AMUE’s managing directors, Stefan Collignon, told me (telephone interview, 28 February 2017).

“The main gist was that we wanted the euro to go through and we were aware that overcharging the project might sink it … There was within AMUE a kind of neo-functional understanding of how monetary union would lead to fiscal and political union. We would have monetary union first and at some point a crisis would force the move to fiscal union too.”^10^

In other words, corporate support for the long-term process of building federal macroeconomic government institutions in the EU has been long-standing and it has been based on some version of the intellectual case for coupling a single currency with fiscal federalism (usually articulated as a version of the theory of optimal currency areas). But practical politics is not based on coherent intellectual designs but rather on opportunity structures that derive from the practical problems that societies and polities confront, and therefore, corporate leaders were happy to sit back and wait for the next crisis to force the issue of fiscal federalism onto the reform agenda.

### Towards fiscal federalism in the EU

The euro had the effect anticipated by its backers. By eliminating currency risk, it brought monetary stability as well as much improved financing conditions for those member states that had built up a reputation for repeated currency devaluations under the EMS. This had been predicted by the Commission’s studies in the early 1990s and it was used as a major argument in favour of the single currency. The argument was that member states with chronic current account deficits and lower per capita output would gain access to greater volumes of cheaper capital that would allow them to increase productive investments and catch-up with the rest of the EU.

The improvement in financing conditions was also driven by a microeconomic development. Financial services corporations had lagged behind their industrial peers in terms of their Europeanization in the 1980s and 1990s. A wave of national consolidations in financial services took place in the decade to 2003 and a number of mega-banks and mega-insurers emerged that began spreading their operations across member state borders (Bayoumi 2017, 37–43). The investment banking and insurance industries were fully Europeanized by the time of the financial crisis in 2008.

Indeed, the decade leading up to 2008 saw an exponential expansion in cross-border financial flows in the EU (Lane 2013). Just like in the past, the deepening of financial integration led to growing imbalances between the Northern member states and their Southern counterparts that could go on being financed for longer. But unlike the benign scenario of such flows financing a quick catch-up process, the flows fuelled credit bubbles instead, in particular in real-estate and consumer credit. In doing so, they became unsustainable and sowed the seeds of the 2010–12 sovereign debt crisis.

But until the beginning of the Greek sovereign debt crisis in 2010, corporate and political leaders were convinced that these imbalances were no longer an issue as they could no longer give rise to currency risk. Consequently, the risk that imbalances could unravel, giving rise to sovereign credit risk, was entirely discounted. As put by Joseph Ackermann, Deutsche Bank CEO in 2002–2012, perceptions were “different before, everybody felt [sovereign bonds were] risk-free assets”. Consequently, he admitted, “the first ten years were so successful that we forgot a little bit to really push for … much more integration … some sort of fiscal union”.^11^

However, the unravelling of the imbalances is precisely what happened from 2010 onwards. The 2008 financial crisis led to a flight to safe havens while in Southern EU member states, the recession of 2008–2009 led to soaring public indebtedness precisely at a time of capital flight. This raised the issue of these member states’ ultimate solvency. When financial investors realized that the Eurozone did not involve a mechanism through which the fiscal liabilities of member states could be collectively backstopped, the flight to safety became a stampede.

This was largely down to the decentralized structure of public finance in the EU. Given the non-existent market for federal public debt, allowing member states to default was not an option because the entire system would be destabilized by calling into question the safe asset status of member state bonds (interview with senior anonymous Commission official, 25 April 2019). Large financial corporations organize their balance sheets (and the whole financial system by extension) around these safe assets. From this point of view, the alternative to backstopping the entire stock of member state bonds would be to transfer a large share of sovereign indebtedness to the federal level, which would allow in principle for sub-federal bankruptcies that would not destabilize the whole system (like in the present-day USA). Given that this alternative was not a realistic prospect in the short-run, backstopping member state bonds was both the corporate-friendly solution and the bridge to such a system in the future.

This created a dilemma for the EU’s political leaders. They could either stick to the original design of the Eurozone whereby the Treaties prohibited fiscal liability mutualization, either through the direct assumption of a member state’s liabilities by the rest of the EU (article 125 TFEU) or through the ECB’s balance sheet (article 123 TFEU). Alternatively, they could allow for some kind of mutualization in order to provide a guarantee to investors that member state debt remained the safe asset around which the European financial system—in particular the EU’s mega-banks’ balance sheets—could be organized. The ultimate decision was therefore about political development towards some form of fiscal federalism.

The initial instinct of political leaders in the Northern member states, anxious to limit the liability of their taxpayers, was to stick with the original design by introducing some kind of debt restructuring mechanism. Investors would thus have to shoulder at least part of the cost of bailing-out member states that had lost market access. In this perspective, sovereign debt markets would be governed by the logic of market discipline and competition among member states to prove their creditworthiness, instead of by the pooling of fiscal liability (telephone interview with Thomas Steffen, German deputy finance minister in 2012–2018, 24 April 2019). This materialized in November 2010 in an agreement struck at the coastal resort of Deauville in Northern France between German chancellor Angela Merkel and French president Nicolas Sarkozy to introduce “private sector involvement” in order to deal with future sovereign debt crises.

However, as I have extensively documented elsewhere (Georgiou 2019), this decision met with the head-on opposition of the corporate community in general and of the financial corporations in particular. Europe’s mega-banks and insurers went on a “credit strike” by unloading their holdings of sovereign debt and refusing to subscribe to new issues of such debt. This precipitated the great speculative crisis on sovereign debt markets of November 2010-September 2012 that became the hallmark of the Eurozone crisis.

The financial corporations’ credit strike finally swayed policymakers when the German government agreed in the summer of 2012 to allow the ECB to step in and provide an unlimited guarantee to investors by promising to buy as many sovereign bonds as necessary to stabilize the situation and eliminate sovereign credit risk. As Waltraud Schelkle (2013) has argued, this decision amounts to the introduction of a “fiscal union by default”, as the balance sheet of the central bank has become the main vehicle for mutualizing fiscal risks in the EU.

That decision epitomizes the broader political decision to move beyond the Eurozone’s initial design towards some kind of fiscal federalism. At the same time that political leaders found a way towards “fiscal integration by default”, they also started debating reforms to move towards plain fiscal federalism with a substantial federal budget. Momentum in that direction petered out after 2013 as the speculative crisis died down and policymakers were not faced with the imminent danger of a collapse of the single currency. What really triggered substantial movement forward was the 2020 public health crisis. The German government’s endorsement of a proposal for the issuance of 750 billion euros in debt by the Commission to fund public investments in decarbonization, digitalization and health signals a sea change in terms of developments towards fiscal federalism. That proposal also received strong backing by corporate Europe (ERT 2020), which very likely played a key role in Berlin’s about-face (Chazan 2020).

To sum up, corporate pressure in favour of federalizing fiscal liability in the EU has set the parameters within which the push towards fiscal federalism in the EU has been taking place over the past ten years. Initially, corporate concerns were with the status of sovereign bonds as safe assets and their own balance sheets. In practice, however, this also entails an interest in the introduction of some kind of federal insurance scheme against asymmetric shocks (this came up in many of the interviews I conducted with corporate bankers and other representatives of employers’ organizations in 2017–2019) through the provision of public goods by the federal level of government.

## Taking stock of the comparison

### A profound similarity

This outline of the relevant historical developments provides support for the claim that there is a profound similarity between the two cases, namely that the functional pressures arising from the corporate reconstruction of capitalism in America and Europe have generated a common trend towards federal political development and macroeconomic government institutions. In both the USA and the European Union, these institutions were created in order to pro-actively stabilize the macroeconomy by pursuing counter-cyclical monetary and fiscal policies, once a large-scale single market structured by large corporations was institutionalized. In both polities, the agents of these functional pressures were the leaders of large corporations, who have had to devise appropriate strategies in order to circumvent political opposition to federal institutional development.

In the USA, the creation of a federal central banking system was a response to the growing financial instability generated by the rise of corporate capitalism within the context of the National Banking System. The creation of an integrated national economy brought about by the corporate economy and in particular of national capital and money markets, aggravated the fault lines in that System. This translated into regular bouts of credit scarcity and runs on the nation’s largest financial institutions. Moreover, given the peculiar character of the American financial system, whereby money and securities markets became tightly interconnected, this fragility also became a source of instability for the rising system of socialized ownership of the large corporations through the stock market. The Federal Reserve System was created to tame this instability by counter-cyclically managing the money supply, acting as a lender of last resort and decoupling securities and money markets.

In the European Union, the creation of a federal central banking system was a response to the growing monetary instability generated by the maturing of corporate capitalism within the context of the European Monetary System based on member state currencies. As large corporations spread their operations across the European market, the growth in cross-border financial flows this gave rise to destabilized the EMS and led to regular runs on weak currencies that wreaked havoc with relative prices and therefore the ability of these pan-European corporations to plan their cross-border activities. Moreover, the speculative pressures on weak currencies regularly led to overly stringent monetary tightening in parts of the European market, which had a stifling effect on growth. The European Central Bank and the single currency were created to simply eliminate the immediate source of the instability, namely the multiplicity of national currencies and allow for a general loosening of monetary policy expected to provide a boost to growth.

In the USA, the rise of the third system of government finance (Wallis 2000) was partly the continuation at the federal level of the secular trend for greater government intervention in the economy to provide public goods necessary for the functioning of the corporate economy (education and skilled labour, health, infrastructure and so on) and partly the result of the corporate demand to stabilize labour and thus product markets in order to tame competition and stabilize the investment system based on the large accumulation of fixed capital. The system also reflects a peculiar conservative version of Keynesianism promoted by corporate leaders whereby inbuilt automatic stabilizers are the main tool of counter-cyclical macroeconomic management.

In the European Union, the trend towards fiscal federalism is ongoing, if not in its infancy. Yet here again, this trend has emerged as a necessary tool for preserving the existing system of corporate finance based on the safe-asset status of sovereign debt, as well as providing some kind of asymmetric shock absorber in order to prevent the instability generated by sharp regional macroeconomic imbalances.

### Important contrasts

However, this profound similarity has found expression in ways that give rise to politically important contrasts. These have to do with the timing as well as the political pattern of the developments examined. In Europe, the process of corporate reconstruction took place within a political structure that was initially extremely fragmented and that lacked even the semblance of a federal charter. In America, a formal federal constitution assigning specific and, in theory, extensive powers to the federal government had been in place for more than a century.

The states that would go on to form the European Union were already powerful bureaucracies presiding over markets of a certain size. Conversely, in the USA, the states were puny institutions with little if no administrative capacities to begin with (remember Skowronek’s thesis about the “state of parties and courts” that was supplanted by the national administrative state at the same time corporate capitalism took hold) and presiding over domestic markets of limited scope.

This initial divergence in political structure acted as an obstacle to the corporate reconstruction of European capitalism, whereas in the USA the process was not slowed down by the initial lack of political centralization. The divergence meant that at least part of the process of corporate reconstruction could take place within the confines of the member states in Europe, as part of an overall structure that remained extremely decentralized politically.

For the purposes of this paper, this has meant that in the European Union, activist central banks and fiscal policies already existed when the pressure to reorganize them at the federal level started exerting itself. In Europe, as a result, creating federal macroeconomic government institutions has mostly been a matter of merging existing national structures and policies. In the field of money, this has entailed replacing national currencies by a single European one and merging the national central banks into a federal organization. In the fiscal field, this has entailed linking together separate national systems of fiscal liability and transferring to the federal level some of the counter-cyclical borrowing and spending hitherto carried out by the member states.

In the USA, on the contrary, the rise of counter-cyclical government intervention in the economy—both in monetary and fiscal policy—was quasi-contemporaneous with its federalization. There had been timid experiments with central reserve city clearing house certificates in the 1890s and early 1900s and even attempts by the Treasury to act as a proto-central bank, but these hardly amounted to a solidly established system of macro-management of the currency. Similarly, the trend towards an expanded fiscal policy to provide public goods necessary for corporate capitalism began at the local and state level in the USA. However, this trend did not get very far before the federal government had to step in and assume the bulk of the growth in public spending, borrowing and fiscal revenue.

The contrasts are important because they point to the historical peculiarities of each case that mean that there is no final destination for the European Union that is bound to resemble the current political structure of the USA. The functional pressures arising from the corporate reconstruction of capitalism do not entail a particular teleology of federal political development. It is, for instance, not possible to make a prediction on the basis of the analysis in this paper about the future shape of fiscal federalism in the EU (how far will the EU budget grow, what new taxes will it rest upon, what spending items will it encompass). However, what the parallels do suggest is that the EU is highly likely to keep moving towards greater fiscal centralization with the active support of the corporate community.

The comparison in this paper has focused on what I claim is a profound similarity, namely the presence of a common independent variable in both cases in the shape of the rise of corporate capitalism within both polities. The paper has consciously neglected other historical factors of fundamental importance such as the legacy political structures and cultures within which corporate capitalism has matured. These clearly exert a defining influence on the respective historical trajectories of the two polities and would need to be properly incorporated in a fully fledged comparative historical examination.

## Footnotes

1 Banking union was the other major reform. This has fiscal implications, but seen as banking policy falls outside the scope of this paper, I will not be discussing it.

2 Here, one major source of inspiration is the American Political Development school (Orren and Skowronek 2004; Valelly, Mettler and Lieberman 2016). My argument is that EU studies should be recast as European Political Development. EU studies evolved out of regional integration theories in the field of IR and they still have an unmistakeable IR bend to them. This is what leads so much scholarship on the Eurozone down the IPE route in search for organizing concepts around which to develop their analysis.

3 I have developed this theoretical framework elsewhere, largely based on Jean-Christophe Defraigne’s (2004) economic history of European integration.

4 I offer no theoretical claim about whether there is a necessary order in the sequence between monetary and fiscal policies. I would tentatively argue that creating conditions of monetary stability takes precedence because in its absence, holding together a system of stable relative prices across geographical regions and sectors in a single market becomes elusive, and therefore, the very premise of a large-scale single market is sapped.

5 For an overall but concise treatment of the historiographical debate on the rise of “big government”, see Sylla (1991). Fusfeld (1972) provides a synthesis based on the already vast literature on corporate capitalism and the state.

6 He further claims that “the reform movement was the preparatory school in which a corporate-industrial business elite worked out a world view or social philosophy appropriate to its constituency’s leadership of a modern society” (Livingston 1986, 18).

7 The long-term incidence of military spending on government growth is negligible. Peaks in military spending occurred at times of major wars but the overall trend continued even when military outlays were scaled back.

8 Herbert Stein’s book is notable in this connection because it is considered the classic study of the rise of Keynesianism in the USA. Stein worked for the CED in 1945–67, before joining Richard Nixon’s Council of Economic Advisers in 1969 (which he presided in 1972–74). He notes (1969, 173) that the CED’s creation in 1942 was an “example of the acceptance by conservatives” of the fiscal revolution.

9 A list of these agencies is available online at https://europa.eu/european-union/about-eu/agencies_en.

10 Davignon broadly confirmed this when I interviewed him in Brussels.

11 Remarks delivered at a speech at the Atlantic Council, Washington D.C. on 4 June 2012. Available at https://www.atlanticcouncil.org/commentary/transcript/the-euro-a-dim-and-dismal-future-with-josef-ackermann-6-4-12-transcript/.
